# Insensibility during Surgical Operations Produced by Inhalation

**Published:** 1847-01

**Authors:** Henry J. Bigelow


*Insensibility during Surgical Operations produced by Inhalation.* By Henry J. Bigelow, M. D.—It may be interesting to know how far medical inhalation has been previously employed. Medical inhalation has been often directed to the amelioration of various pulmonary affections, with indifferent success. Instruments called *inhalers* were employed long ago by Mudge, Gardiner and Darwin, and the apparatus fitted up by Dr. Beddoss and Mr. James Watt, for respiring various gases, has given birth to some octavo volumes. More recently Sir Charles Scudamore has advocated the inhalation of iodine and conium in phthisis, and the vapor of tar has been often inhaled in the same disease. The effects of stramonium, thus administered, have been noticed by Sigmond.

*The inhalation of the ethers* has been recommended in various maladies, among which may be mentioned phthisis and asthma. "On sait que la respiration de l'ether sulfurique calme souvent les accidents nerveux de certains croup," is from the Dict. des Sc. Med.; but I find that mention of the inhalation of this agent is usually coupled with a caution against its abuse, grounded apparently upon two or three cases, quoted and re-quoted. Of these the first is from Brande's Journal of Science, where it is thus reported: "By imprudent respiration of sulphuric ether, a gentleman was thrown into a very lethargic state which continued from one to three hours, with occasional intermissions and great depression of spirits—the pulse being for many days so low that considerable fears were entertained for his life." Christison quotes the following from the Midland Med. and Surg. Journal, to prove that nitric ether in vapor is a dangerous poison when too freely and too long inhaled: "A druggist's maid servant was found one morning dead in bed, and death had evidently arisen from the air of her apartment having been accidentally loaded with vapor of nitric ether, from the breaking of a three gallon jar of the Spiritus Æth. Nitric. She was found lying on her side, with her arms folded across her chest, the countenance and posture composed, and the whole appearance like a person in a deep sleep. The stomach was red internally and the lungs were gorged." The editor of the journal where this case is related, says he is acquainted with a similar instance, where a young man was found completely insensible from breathing air loaded with *sulphuric ether*, remained epileptic for some hours, and would undoubtedly have perished had he not been discovered and removed in time. Ether is now very commonly administered internally as a diffusible stimulant and antispasmodic, in a dose of one or two drachms. But here also we have the evidence of a few experiments that ether is capable of producing grave results under certain circumstances. Orfila killed a dog by confining a small quantity in the stomach by means of a ligature around the œsophagus. Jager found that ℥ ss. acted as a fatal poison on a crane. It was for a long time supposed to be injurious to the animal economy. The old Edinburg Dispensatory, republished here in 1816, explicitly states that it is to be inhaled by holding in the mouth a piece of sugar containing a few drops, and also that regular practitioners give only a few drops for a dose; "though," it adds, "empirics have sometimes ventured upon much larger quantities, and with incredible benefit." p. 566. Nevertheless, it was known to have been taken in correspondingly large doses with impunity. The chemist Bucquet, who died of scirrhus of the colon, with inflammation of the stomach and intestines, took before his death a pint of ether daily, to alleviate his excruciating pains, (he also took 100 gr. opium daily;) and Christison mentions an old gentleman who consumed for many years ℥ xvi. every eight or ten days. Such facts probably led Merat and De Lens, in their Matiere Medicale, to question its grave effects when swallowed. Mentioning the case of Bucquet, they say, even of its inhalation, that it produces only "un sentiment de frachieur que suit bientôt une legère excitation."

This variety of evidence tends to show that the knowledge of its effects, especially those of its inhalation, was of uncertain character. Anthony Todd Thompson well sums up what I conceive to have been the state of knowledge at the time upon this subject, in his London Dispensatory of 1818. "As an antispasmodic, it relieves the paroxysm of spasmodic asthma, whether it be taken into the stomach, or its vapor only be inhaled into the lungs. Much caution, however, is required in inhaling the vapor of ether, as the imprudent inspiration of it has produced lethargic and apoplectic symptoms." In his Materia Medica and Therapeutics, of 1832, however, omitting all mention of inhalation, he uses the following words: "Like other diffusible excitants, its effects are rapidly propa gated over the system, and soon dissipated. From its volatile nature its exciting influence is probably augmented; as it produces distension of the stomach and bowels, and is thus applied to every portion of their sensitive surface. It is also probable that it is absorbed in its state of vapor, and is therefore directly applied to the nervous centres. It is the diffusible nature of the stimulus of ether which renders it so well adapted for causing sudden excitement, and producing immediate results. Its effects, however, so soon disappear, that the dose requires to be frequently repeated."

Nothing is here said of inhalation, and we may fairly infer that the process had so fallen into disrepute, or was deemed to be attended with such danger, as to render a notice of it superfluous in a work treating, in 1832, of therapeutics.

It remains briefly to describe the process of inhalation by the new method, and to state some of its effects. A small two-necked glass globe contains the prepared vapor, together with sponges to enlarge the evaporating surface. One aperture admits the air to the interior of the globe, whence, charged with vapor, it is drawn through the second into the lungs. The inspired air thus passes through the bottle, but the expiration is diverted by a valve in the mouth piece, and escaping into the apartment is thus prevented from visiting the medicated vapor. A few of the operations in dentistry, in which the preparation has as yet been chiefly applied, have come under my observation. The remarks of the patients will convey an idea of their sensations.

A boy of sixteen, of medium stature and strength, was seated in the chair. The first few inhalations occasioned a quick cough, which afterwards subsided; at the end of eight minutes the head fell back, and the arms dropped, but owing to some resistance in opening the mouth, the tooth could not be reached before he awoke. He again inhaled for two minutes, and slept three minutes, during which time the tooth, an inferior molar, was extracted. At the moment of extraction the features assumed an expression of pain, and the hand was raised. Upon coming to himself he said he had had a first-rate dream—very quiet," he said, "and had dreamed of Napoleon—had not the slightest consciousness of pain—the time had seemed long;" and he left the chair, feeling no uneasiness of any kind, and evidently in a high state of admiration. The pupils were dilated during the state of unconsciousness, and the pulse rose from 130 to 142.

A girl of 16 immediately occupied the chair. After coughing a little, she inhaled during three minutes, and fell asleep, when a molar tooth was extracted, after which she continued to slumber tranquilly during three minutes more. At the moment when force was applied she flinched and frowned, raising her hand to her mouth, but said she had been dreaming a pleasant dream, and knew nothing of the operation.

A stout boy of 12, at the first inspiration coughed considerably, and required a good deal of encouragement to induce him to go on. At the end of three minutes from the first fair inhalation, the muscles were relaxed and the pupil dilated. During the attempt to force open the mouth he recovered his conciousness, and again inhaled during two minutes, and in the ensuing one minute two teeth were extracted, the patient seeming somewhat conscious, but upon actually awaking he declared "it was the best fun he ever saw," avowed his intention to come there again, and insisted upon having another tooth extracted upon the spot. A splinter which had been left, afforded an opportunity of complying with his wish, but the pain proved to be considerable. Pulse at first 110, during sleep 96, afterwards 144; pupils dilated.

The next patient was a healthy-looking, middle-aged woman, who inhaled the vapor for four minutes; in the course of the next two minutes a back tooth was extracted, and the patient continued smiling in her sleep for three minutes more. Pulse 120, not affected at the moment of the operation, but smaller during sleep. Upon coming to herself, she exclaimed that "it was beautiful—she dreamed of being at home—it seemed as if she had been gone a month," These cases, which occurred successively in about an hour, at the room of Dr. Morton, are fair examples of the average results produced by the inhalation of the vapor, and will convey an idea of the feelings and expressions of many of the patients subjected to the process. Dr. Morton states that in upwards of two hundred patients, similar effects have been produced. The inhalation, after the first irritation has subsided, is easy, and produces complete unconsciousness at the expiration of a period varying from two to five or six, sometimes eight minutes; its duration varying from two to five minutes, during which the patient is completely insensible to the ordinary tests of pain. The pupils of the cases I have observed have been generally dilated; but with allowance for excitement and other disturbing influences, the pulse is not affected, at least in frequency; the patient remains in a calm and tranquil slumber, and wakes with a pleasurable feeling. The manifestation of consciousness or resistance I at first ascribed to the reflex function, but I have since had cause to modify this view.

It is natural to enquire whether no accidents have attended the employment of a method so wide in its application, and so striking in its results. I have been unable to learn that any serious consequences have ensued. One or two robust patients have failed to be affected. I may mention as an early and unsuccessful case, its administration in an operation performed by Dr. Hayward, where an elderly woman was made to inhale the vapor for at least half an hour without effect. Though I was unable at the time to detect any imperfection in the process, I am inclined to believe that such existed. One woman became excited, and required to be confined to the chair. As this occurred to the same patient twice, and in no other case as far as I have been able to learn, it was evidently owing to a peculiar susceptibility. Very young subjects are affected with nausea and vomiting, and for this reason Dr. M. has refused to administer it to children. Finally, in a few cases, the patient has continued to sleep tranquilly for eight or ten minutes, and once, after a protracted inhalation, for the period of an hour.

The following case, which occurred a few days since, will illustrate the probable character of future accidents. A young man was made to inhale the vapor, while an operation of limited extent, but somewhat protracted duration, was performed by Dr. Dix upon the tissues near the eye. After a good deal of coughing the patient succeeded in inhaling the vapor, and fell asleep at the end of about ten minutes. During the succeeding two minutes the first incision was made, and the patient awoke, but unconscious of pain. Desiring to be again inebriated, the tube was placed in his mouth and retained there about twenty-five minutes, the patient being apparently half affected, but, as he subsequently stated, unconscious. Respiration was performed partly through the tube, and partly with the mouth open. Thirty-five minutes had now elapsed, when I found the pulse suddenly diminishing in force, so much so that I suggested the propriety of desisting. The pulse continued decreasing in force, and from 120 had fallen to 96. The respiration was very slow, the hands cold, and the patient insensible. Attention was now of course directed to the return of respiration and circulation. Cold affusions as directed for poisoning with alcohol, were applied to the head, the ears were syringed, and ammonia presented to the nostrils and administered internally. For fifteen minutes the symptoms remained stationary, when it was proposed to use active exercise, as in case of narcotism from opium. Being lifted to his feet, the patient soon made an effort to move his limbs, and the pulse became more full, but again decreased in the sitting posture, and it was only after being compelled to walk during half an hour that the patient was able to lift his head. Complete consciousness returned only at the expiration of an hour. In this case the blood was flowing from the head, and rendered additional loss of blood unnecessary. Indeed the probable hemorrhage was previously relied on as salutary in its tendency.

Two recent cases serve to confirm, and one, I think, to decide the great utility of this process. On Saturday, the 7th Nov., at the Mass. General Hospital, the right leg of a young girl was amputated above the knee, by Dr. Hayward, for disease of this joint. Being made to inhale the preparation, after protesting her inability to do so from the pungency of the vapor, she became insensible in about five minutes. The last circumstance she was able to recall was the adjustment of the mouth-piece of the apparatus, after which she was unconscious until she heard some remark at the time of securing the vessels—one of the last steps of the operation. Of the incision she knew nothing, and was uhable to say, upon my asking her, whether or not the limb had been removed. She refused to answer several questions during the operation, and was evidently completely insensible to pain or other external influences. This operation was followed by another, consisting of the removal of a part of the lower jaw, by Dr. Warren. The patient was insensible to the pain of the first incision, though she recovered her consciousness in the course of a few minutes.

The character of the lethargic state, which follows this inhalation, is peculiar. The patient loses his individuality and awakes after a certain period, either entirely unconscious of what has taken place, or retaining only a faint recollection of it. Severe pain is sometimes remembered as being of a dull character; sometimes the operation is supposed by the patient to be performed on somebody else. Certain patients, whose teeth have been extracted, remember the application of the extracting instruments; yet none have been conscious of any real pain.

As before remarked, the phenomena of the lethargic state are not such as to lead the observer to infer this insensibility. Almost all patients under the dentist's hands scowl or frown; some raise the hand. The patient whose leg was amputated, uttered a cry when the sciatic nerve was divided. Many patients open the mouth, or raise themselves in the chair, upon being directed to do so. Others manifest the activity of certain intellectual faculties. An Irishman objected to the pain, that he had been promised an exemption from it. A young man taking his seat in the chair and inhaling a short time, rejected the globe, and taking from his pockets a pencil and card, wrote and added figures. Dr. M. supposing him to be affected, asked him if he would now submit to the operation, to which the young man willingly assented. A tooth was accordingly extracted, and the patient soon after recovered his senses. In none of these cases had the patients any knowledge of what had been done during their sleep.

I am, as yet, unable to generalize certain other symptoms to which I have directed attention. The pulse has been, as far as my observation extends, unaltered in frequency, though somewhat diminished in volume, but the excitement preceding an operation, has, in almost every instance, so accelerated the pulse that it has continued rapid for a length of time. The pupils are in a majority of cases dilated; yet they are in certain cases unaltered, as in the above case of amputation.

The duration of the insensibility is another important element in the process. When the apparatus is withdrawn at the moment of unconsciousness, it continues, upon the average, two or three minutes, and the patient then recovers completely or incompletely, without subsequent ill effects. In this sudden cessation of the symptoms, this vapor in the air tubes differs in its effects from the narcotics or stimulants in the stomach, and, as far as the evidence of a few experiments of Dr. Morton goes, from the ethereal solution of opium when breathed. Lassitude, headache and other symptoms lasted for several hours, when this agent was employed.

But if the respiration of the vapor be prolonged much beyond the first period, the symptoms are more permanent in their character. In one of the first cases, that of the young boy, the inhalation was continued during the greater part of ten minutes, and the subsequent narcotism and drowsiness lasted more than an hour. In a case alluded to before, the narcotism was complete; during more than twenty minutes, the insensibility approached to coma.

Such cases resemble those before quoted from Christison and other authors, and show that the cessation of the inhalation, after it has been prolonged for a length of time, does not produce a corresponding cessation of the symptoms; while, if the inhalation is brief, the insensibility ceases in a short time. Recovery, in the latter case, is not improbably due to the complete and rapid elimination of the vapor from the lungs; the

7

more gradual return of consciousness, in the former case, to the presence of a larger quantity of unexhaled particles. A fact mentioned by Christison bears upon this point. This author states that insensibility from the presence of a large quantity of alcohol in the stomach, often gives place to a complete and sudden return of consciousness, when the alcohol is removed by the stomach pump. It is probable that the vapor of the new preparation ceases early to act upon the system, from the facility with which it is exhaled.

The process is obviously adapted to operations which are brief in their duration, whatever be their severity. Of these, the two most striking are, perhaps, amputations and extraction of teeth. In protracted dissections, the pain of the first incision alone is of sufficient importance to induce its use; and it may hereafter prove safe to administer it for a length of time, and to produce a narcotism of an hour's duration. It is not unlikely to be applicable in cases requiring a suspension of muscular action; such as the reduction of dislocations or of strangulated hernia: and finally it may be employed in the alleviation of functional pain, of muscular spasm, as in cramp and colic, and as a sedative or narcotic.

The application of the process to the performance of surgical operations, is, it will be conceded, new. If it can be shown to have been occasionally resorted to before, it was only an ignorance of its universal application and immense practical utility that prevented such isolated facts from being generalized.—*Boston Med. Jour.*

